# Gonadotropin elevation is ootoxic to ovulatory oocytes and inhibits oocyte maturation, and activin decoy receptor ActRIIB:Fc therapeutically restores maturation

**DOI:** 10.1186/s12958-024-01224-8

**Published:** 2024-05-06

**Authors:** Lori R. Bernstein, Amelia C. L. Mackenzie, Charles L. Chaffin, Se-Jin Lee, Duane C. Kraemer, Istvan Merchenthaler

**Affiliations:** 1 Pregmama, LLC, Gaithersburg, MD 20886 USA; 2 Department of Cell Biology and Genetics, Texas A & M School of Medicine, College Station, TX 77843 USA; 3 Department of Epidemiology and Public Health, University of Maryland School of Medicine, Baltimore, MD 21201 USA; 4 Department of Veterinary Integrative Biosciences, Texas A&M School of Veterinary Medicine, College Station, TX 77843 USA; 5 FHI 360, Durham, NC 27701 USA; 6 Obstetrics, Gynecology & Reproductive Sciences, University of Maryland School of Medicine, Baltimore, MD 21201 USA; 7 University of Connecticut School of Medicine, Farmington, CT 06030 USA; 8 The Jackson Laboratory for Genomic Medicine, Farmington, CT 06030 USA; 9 Department of Veterinary Physiology and Pharmacology, Texas A & M School of Veterinary Medicine, College Station, TX 77843 USA; 10 Department of Anatomy and Neurobiology, University of Maryland School of Medicine, Baltimore, MD 21201 USA

**Keywords:** eCG, FSH, Ootoxicity, Oocyte maturation, ActRIIB:Fc, SAMP8 mice, Fertility, Advanced maternal age (AMA), FSH OoToxicity Hypothesis: FOOT Hypothesis

## Abstract

**Background:**

Elevated FSH often occurs in women of advanced maternal age (AMA, age ≥ 35) and in infertility patients undergoing controlled ovarian stimulation (COS). There is controversy on whether high endogenous FSH contributes to infertility and whether high exogenous FSH adversely impacts patient pregnancy rates.

**Methods:**

The senescence-accelerated mouse-prone-8 (SAMP8) model of female reproductive aging was employed to assess the separate impacts of age and high FSH activity on the percentages (%) of viable and mature ovulated oocytes recovered after gonadotropin treatment. Young and midlife mice were treated with the FSH analog equine chorionic gonadotropin (eCG) to model both endogenous FSH elevation and exogenous FSH elevation. Previously we showed the activin inhibitor ActRIIB:Fc increases oocyte quality by preventing chromosome and spindle misalignments. Therefore, ActRIIB:Fc treatment was performed in an effort to increase % oocyte viability and % oocyte maturation.

**Results:**

The high FSH activity of eCG is ootoxic to ovulatory oocytes, with greater decreases in % viable oocytes in midlife than young mice. High FSH activity of eCG potently inhibits oocyte maturation, decreasing the % of mature oocytes to similar degrees in young and midlife mice. ActRIIB:Fc treatment does not prevent eCG ootoxicity, but it restores most oocyte maturation impeded by eCG.

**Conclusions:**

FSH ootoxicity to ovulatory oocytes and FSH maturation inhibition pose a paradox given the well-known pro-growth and pro-maturation activities of FSH in the earlier stages of oocyte growth. We propose the FOOT Hypothesis (“FSH OoToxicity Hypothesis), that FSH ootoxicity to ovulatory oocytes comprises a new driver of infertility and low pregnancy success rates in DOR women attempting spontaneous pregnancy and in COS/IUI patients, especially AMA women. We speculate that endogenous FSH elevation also contributes to reduced fecundity in these DOR and COS/IUI patients.

Restoration of oocyte maturation by ActRIB:Fc suggests that activin suppresses oocyte maturation *in vivo.* This contrasts with prior studies showing activin A promotes oocyte maturation *in vitro*. Improved oocyte maturation with agents that decrease endogenous activin activity with high specificity may have therapeutic benefit for COS/IVF patients, COS/IUI patients, and DOR patients attempting spontaneous pregnancies.

**Supplementary Information:**

The online version contains supplementary material available at 10.1186/s12958-024-01224-8.

## Background

Only a competent oocyte that is viable, mature, and euploid can make a healthy baby [1]. Dying and dead oocytes do not develop. Immature oocytes cannot be fertilized, and aneuploid oocytes usually do not implant, survive, or make healthy offspring. Previously we reported that senescence-accelerated mouse-prone-8 (SAMP8) female mice emulate multiple attributes of reproductive aging that occur in women with increasing age. These include shorter reproductive cycles, higher endogenous follicle stimulating hormone (FSH) levels, more variability of FSH levels between consecutive cycles, increased predisposition to oocyte aneuploidy, and diminished fertility compared to younger mice [2, 3]. Recently, Polonio et al. showed that telomeres in ovaries of SAMP8 mice are shorter than the those of age-matched midlife control mice of the senescence-resistant strain SAMR1 [4]. Viability of ovarian SAMP8 oocytes was lower than SAMR1 oocytes after gonadotropin stimulation.

Here, the SAMP8 model was employed to test the separate impacts of age and FSH activity on percentage (%) of ovulated oocytes that are viable and the % that are mature. High FSH activity significantly reduces the % of ovulated oocytes that are viable, especially in midlife mice, and potently reduces the % of ovulated oocytes that are mature, both in midlife and young mice. We therefore sought to therapeutically improve rates of SAMP8 oocyte viability and maturity.

Activins belong to the transforming growth factor β (TGFβ) superfamily of regulatory signaling ligands. TGFβ superfamily proteins play critical functions mediating development and differentiation, metabolism, homeostasis, immunity, inflammation, and disease processes in multiple tissues and organ systems. The TGFβ superfamily is primarily comprised of the TGFβ’s, activins, inhibins, Growth and Differentiation Factors (GDFs), Nodals, and the Bone Morphogenic Proteins (BMPs; [5], and references therein). A dimeric ligand of the TGFβ superfamily binds to a dimeric Type I subunit plus a dimeric Type II subunit to generate the functional hexameric ligand-receptor serine/threonine kinase protein complex. This complex initiates a signal transduction cascade that causes phosphorylation of SMAD family transcription factors, which translocate to the nucleus and transactivate expression of target genes to execute biological responsiveness.

Diverse activin and inhibin species are generated by dimerization of α and β subunits. The activin receptor binding species are Activin A (β_A_β_A_), activin AB (β_A_β_B_), activin B (β_B_β_B_), inhibin A (αβ_A_), and inhibin B (αβ_B_). Type 1 activin receptors are ACVR1, ACVR1B, and ACVR1C, and Activin Type II receptors are ActRIIA (alias ACVR2A) and ActRIIB (alias ACVR2B) [6]. Several isoforms of activin and inhibin mRNAs and proteins are expressed in granulosa and thecal cells, and several activin and inhibin protein isoforms are found in follicular fluid [7]. Type II activin receptors ActRIIA and ActRIIB mRNAs are expressed in follicular cells and in oocytes during oocyte maturation [8] and references therein; [9]. They are also expressed in fallopian tube, endometrium, and placenta. Activin ligands and their receptors in the ovary modulate oocyte and follicular development, growth, ovulation, and corpus luteum function. Activins and their receptors in the pituitary modulate the hypothalamic-pituitary ovarian (HPO) axis, with activin upregulating, and inhibin downregulating pituitary FSH secretion [6, 7].

Follistatin is a potent endogenous ovarian inhibitor of activin signaling [10–12]. Two follistatin molecules bind activin, encircling it to block its binding to the activin receptor [13]. Therefore in theory, follistatin could be used as a specific reagent to probe involvement of activins in specific biological processes. However, the half-life of follistatin is very short, making administration of follistatin of limited practical value. Activin decoy receptor molecules with long half-lives were therefore devised that bind and sequester activin to neutralize its biological activity. The soluble AcRIIB receptor ActRIIB:Fc (alias ACVR2B:Fc) developed by Lee et al. is one such decoy receptor molecule [14]. It consists an ActRIIB receptor binding domain fused a sequence encoding the Fc region of the IgG molecule.

The Fc domain markedly increases the half-life of the fusion protein. In previous studies we showed that ActRIIB:Fc increases yields of viable ovulated oocytes/mouse in midlife SAMP8 mice, therapeutically prevents oocyte chromosome and spindle misalignments that predispose to aneuploidy, and increases fertility [3]. We therefore administered ActRIIB:Fc to midlife mice to test the notion that it might therapeutically restore viability and/or maturation rates of ovulatory oocytes in eCG-treated midlife SAMP8 mice.

## Methods

### Materials

Supplies and reagents used in the study are as described [2, 3, 15].

### SAMP8 mouse colony

SAMP8 mouse breeding colonies were established and maintained in the animal facilities at the University of Maryland School of Medicine (Baltimore, MD, USA) and Texas A & M School of Veterinary Medicine (College Station, TX, USA).

SAMP8 female mice require a strict protocol for caging density, open air cages, and interspersed male cages to display regular estrous cyclicity. Caging and monitoring of estrus regularity by vaginal smearing and cytology were performed as described [2]. Young SAMP8 mouse age groups were 2.3 – 3.2 months of age (mean 2.7 months), and midlife mouse age groups were 6.5 – 8.7 months of age (mean 7.0 months) at the time of oocyte retrieval.

### eCG treated vs. untreated young and midlife SAMP8 mice

Treatment of mice with the FSH analog equine chorionic gonadotropin (eCG) was administered rather than FSH because the longer half-life of eCG made the investigation financially and logistically feasible.

eCG treatment was comprised of a single IP injection of 5 IU eCG followed 48 h later by IP injection of 5 IU human chorionic gonadotropin (hCG). Control eCG-untreated mice were regularly cycling mice that were not injected with eCG. They were injected with hCG the afternoon of proestrus.

### Oocyte collection and scoring for viability, polar body extrusion, and postovulatory aging

Freshly ovulated ampullary oocytes were collected 14 – 16 h post hCG and visualized in light microscopy as described [2]. Oocytes surrounded by a viable cumulus layer as cumulus-oocyte complexes (COCs) were scored as viable. Dead, degenerated, denuded, and fragmented oocytes were scored as non-viable. COCs with abnormally expanded cumulus were scored as postovulatory aged (POA) by criteria of Hammitt et al. [16].

### Scoring for oocyte meiotic phases

Freshly recovered ovulated oocytes were examined by light microscopy and scored for the presence (+ PB1) or absence (-PB1) of an extruded polar body, a defining characteristic of maturity in the oocyte [17]. Oocytes were fixed, microtubules and chromosomes were fluorescently stained, and examined by fluorescence microscopy as described [2]. Oocytes were scored as GV, GVBD, Pro-metaphase, metaphase I (MI), anaphase, telophase, or metaphase II (MII) according to criteria described [2, 18].

### ActRIIB:Fc preparation

The mouse IgG Fc domain was fused to the extracellular domain of the mouse activin Type II receptor ActRIIB to generate the recombinant ActRIIB:Fc fusion gene sequence [14]. Recombinant ActRIIB:Fc protein was expressed in CHO cells and purified from conditioned media, as described [14].

### Treatment of midlife SAMP8 mice with ActRIIB:Fc, eCG and hCG

SAMP8 mice underwent daily vaginal smearing and cytology [2]. Those with confirmed regular cycles were injected IP with 7 mg/kg ActRIIB:Fc, followed by booster injections every 3 – 4 days to maintain ActRIIB:Fc levels, as described [3]. Ongoing daily vaginal smearing and cytology confirmed that mice maintained regular estrous cyclicity during ActRIIB:Fc treatment [3]. Four midlife test groups were analyzed for oocyte viability and maturation after treatment with ActRIIB:Fc or ActRIIB:Fc + eCG: (1) control (“M-”); (2) eCG-treated (“eCG”); (3) ActRIIB:Fc treated (“Act-R”); (4) ActRIIB:Fc + eCG treated (“Act-R + eCG”). Mice in the M- group were administered vehicle control saline by IP injections every 3–4 days for 18 -23 days until the morning of proestrus, and then administered hCG to induce ovulation. Mice in the eCG group were given 5 IU eCG followed by 5 IU hCG 48 h later. ActRIIB:Fc-treated mice given ActRIIB:Fc were subdivided into the Act-R and Act-R + eCG test groups. Mice in the Act-R group were given hCG IP the morning of proestrus 18 -23 days after the initial injection of ActRIIB:Fc. Mice in the Act-R + eCG group were given 5 IU IP eCG 16–21 days after initial their ActRIIB:Fc initial injection, followed by hCG 48 h post eCG. Thus, both ActRIIB:Fc-treated groups received 19 – 24 days ActRIIB:Fc before oocyte retrieval. Oocytes were harvested from ovarian ampullae 14–16 h after eCG injection.

### Measurement of FSH levels in ActRIIB:Fc-treated midlife mice

Blood sera were prepared from blood collected from regularly cycling midlife saline control- and ActRIIB:Fc-treated mice on the morning of estrus, as described [2]. Sera from mice treated with ActRIIB:Fc for 0 or 1–4 days were from survival bleeds, and sera from mice treated with ActRIIB:Fc for 21 – 24 days were from terminal bleeds after hCG ovulation induction. Sera from each mouse were analyzed in duplicate or triplicate. FSH levels were then averaged to generate a single value for FSH in pg/ml for each mouse. These average values were then averaged within each test group to compute mean FSH/mouse.

### Statistical analyses

Statistical tests were performed to compute *P* values using GraphPad Prism software versions 9.5.1 or 10.0.0 [19] *P* < 0.05 was defined as statistically significant, unless stated otherwise.

### Percentages of oocytes

Overall P values comparing percentages (%) of ovulated viable oocytes, % of oocytes with an extruded polar body (i.e., metaphase II (MII)), and % of oocytes at each maturation phase were computed using Chi-square tests. Pairwise comparisons between test groups were made with 2-tailed Fisher exact tests. *P* values for pairwise comparisons for datasets comprised of > 2 study groups were assessed by the method of Benjamini-Hochberg (B-H; [20]) with a False Discovery Rate of α = 0.05, using a spreadsheet from “The Handbook of Biological Statistics” [21].

### Correlations between % viable and % mature oocytes

R and *P* values for correlation of % viable oocytes and % oocytes with a PB1 were computed by two-tailed Spearman analyses.

### Viable oocytes/mouse, non-viable oocytes/mouse and total oocytes/mouse

Count data were analyzed by Anderson–Darling tests and Levene’s tests (performed with DataPlot: [22]), revealing non-normal distributions and unequal variances among test groups. Brunner, Dette and Munk (BDM) rank-based non-parametric 2-way ANOVA analyses were performed that accommodate count data, non-normally distributed data with unequal variances, ties, and unequal sample sizes [23, 24]. The bdm2way function was employed using the R Statistical program [25] to compute two-factor ANOVA comparisons, and the Wilcox R BDM function was used to compute the one-factor com-parisons [24]. The Wilcox R BDM method is more accommodating for datasets with a high percentage of zeros than other methods, such as a rank-based Bootstrap test [24].

*Percentage of oocytes/mouse that are not viable*. *P* values were computed employing 2-way ANOVA tests comparing the arcsin square root values for mean % yields of non-viable oocytes/mouse. Tukey’s post-tests were used to compute *P* values for 1-Factor comparisons.

### Serum FSH concentrations

Estrus FSH levels in 0, 1–4, and 21–24 day ActRIIB:Fc-treated test groups had normal distributions by the Anderson–Darling test, and had equal variances by Bartlett’s and Levene’s tests (*P* > 0.05; [21, 26]). Thus, overall significance of differences was computed using an Ordinary One-Way ANOVA test, and multiple comparisons between individual test groups were calculated using a Tukey’s multiple comparisons test.

## Results and discussion

### Gonadotropin administration reduces viability of ovulated oocytes in midlife more than young mice

**Fig. 1:**
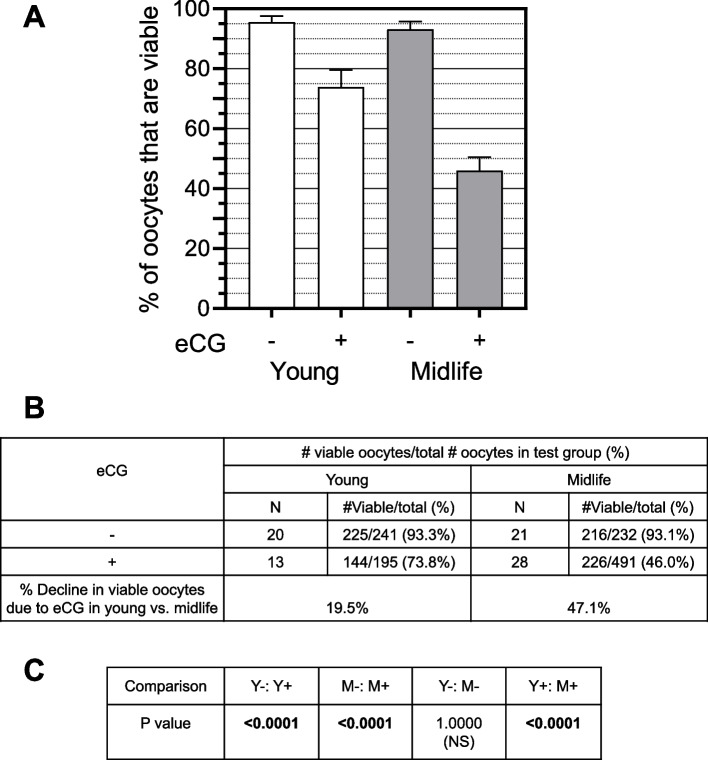
eCG reduces the percentage of ovulated oocytes that are viable in midlife mice more than young mice. **A** *Graphical comparisons*. White bars: young; gray bars: midlife. **B** *Numerical comparisons*. Incremental % decline in viable oocytes due to eCG treatment was computed by subtracting the % viable oocytes after eCG treatment from the % viable oocytes without eCG treatment. **C** *P values for comparisons of #viable/# total oocytes between test groups*. For this and subsequent figures: Y, young untreated; Y + , young eCG treated; M-, midlife untreated; M + , midlife eCG treated. Error bars in graphs are ± 95% CI unless otherwise noted, *N* = number of mice in a test group, *P* values in bold are significant, and *P* values not in bold are not significant (NS)

Both eCG and age had significant impacts on the % of oocytes that are viable in the four test groups of mice (Y-, Y + , M-, M + ; *P* < 0.0001). While there is no significant difference in the % viable oocytes between young and midlife untreated mice (*P* = 1.0000), eCG reduces the % of viable ovulated oocytes from 93.3% to 72.8% in young mice (*P* < 0.0001), and from 93.1% to 46.0% (*P* < 0.0001) in midlife mice (Fig. 1 A-C). The 46.0% of oocytes/mouse that were viable after eCG in midlife is significantly lower than the 73.8% that of oocytes after eCG in young (*P* < 0.0001). A prior study by Goh et al. showed an increase in the combined percentage of degenerated oocytes and embryos in rats after eCG treatment [26]. Studies by Gray and Christman and by Kalthur et al. found that eCG treatments increased oocyte fragmentation rates in mice [27, 28]. However, the current paper is the first report showing that midlife animals are more susceptible than young animals to reductions in % viability caused by elevated FSH activity.

Non-viable oocytes in the four groups were subcategorized as dead, fragmented (i.e., apoptotic), or denuded. 97.0% were dead or apoptotic and only 3.0% were denuded, with no significant pairwise differences in proportions of these subcategories of non-viable oocytes among the four test groups (*P* > 0.05). These data demonstrate that eCG is potently ootoxic to ovulatory oocytes, and ovulatory oocytes of midlife mice are more susceptible to eCG ootoxicity than those of young mice.

Fertility is a function of the number of viable oocytes/mouse that are ovulated. eCG does not affect the number of viable ovulated oocytes/mouse in young mice (viable oocytes/mouse: Y-, 11.25 vs. Y + , 11.08, *P* = 0.9740 (NS)) but it significantly reduces the yield of viable oocytes/mouse in midlife mice (M-: 10.29 vs. M + : 8.07, *P* = 0.0003; [15]). This is 3.07 oocytes/mouse fewer than the number of viable oocytes per mouse ovulated in the young mice, a significant decline with age that would be expected to result in lower fertility/litter sizes in the eCG-treated midlife mice (Y + vs. M + , 11.08 vs. 8.07, *P* = 0.0325; [15]). Given that the *total* oocytes/mouse ovulated after eCG treatment is not significantly different between young and midlife mice (Y + : 15.0/mouse; M + , 17.54/mouse, *P* = 0.2062 (NS), and given that the proportion of total oocytes/mouse that are not viable is greater in midlife than young eCG-treated mice (Y + , 24.5%. vs. M + , 50.5%, *P* = 0.0001), it is evident that the number of viable oocytes/mouse is reduced in midlife mice because eCG is killing more ovulatory oocytes/mouse in midlife than young mice. This is expected to cause a significantly greater decline in fecundability of oocytes from midlife than young mice.

It is not known whether the dead and dying oocytes retrieved from eCG-treated mice were already non-viable when they were ovulated, or started dying while in transit in the ampulla. The latter might suggest that the fallopian tube plays an active role mediating death of oocytes after ovulation under conditions of high FSH. FSH receptors have been found in the oviducts of porcine, bovine, and human, but the full scope of their functions is yet to be elucidated [29–31]. Future studies will determine whether tubal FSH receptors mediate death of ova, and where along the length of the tube this might be occurring.

### Gonadotropin treatment impedes oocyte maturation in young and midlife mice

**Fig. 2:**
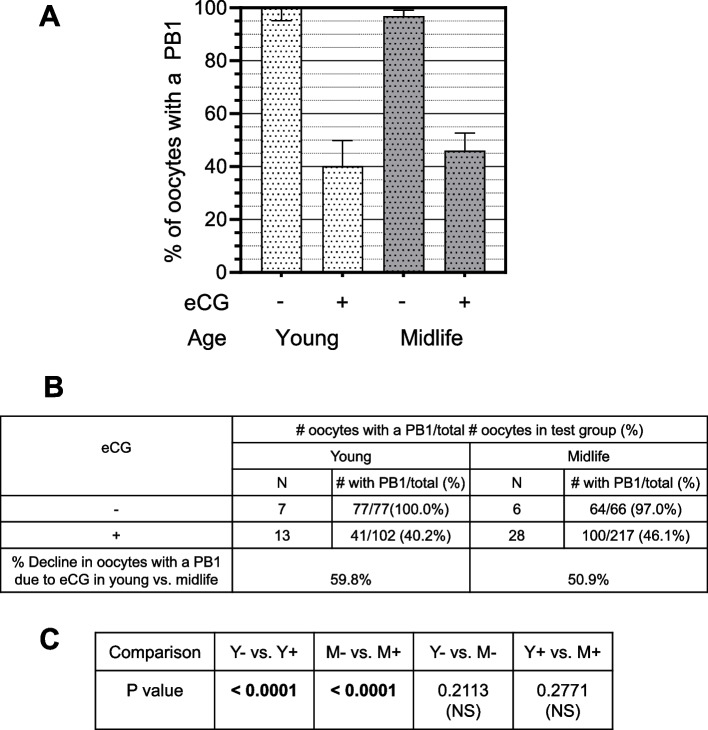
eCG reduces the % of oocytes that mature to MII in young and midlife, with no significant difference with age. **A** *Graphical comparisons of maturation rates*. “- “: eCG untreated; “ + ” eCG-treated. White stippled bars: Young; Gray stippled bars: Midlife. **B **Percent decline in mature oocytes due to eCG treatment. % decline in oocytes with a PB1 due to eCG treatment (fourth row of table) was computed by subtracting the % mature oocytes after eCG treatment from the % mature oocytes without eCG treatment. **C** *P values for comparisons of # mature/# total oocytes between test groups*. Y, young untreated; Y + , young eCG treated; M-, midlife untreated; M + , midlife eCG treated. P values in bold are significant. P values not in bold, not significant

Overall, the adverse effect of eCG on the % of mature oocytes is highly significant (*P* < 0.0001). eCG decreases the % of oocytes that achieve maturity in both young and midlife groups. As for the viability results above, there is no significant difference in the % of mature ovulated oocytes in untreated young vs. midlife mice (Y-, 100% mature vs. M-, 97.0% mature (*P* = 0.2113 (NS); Fig. 2). eCG markedly reduces the % of mature ovulated oocytes from young mice at the time of retrieval, from 100% to 40.2% (*P* < 0.0001). eCG reduces the % of mature oocytes from midlife mice: from 97.0% to 46.0% (M + : *P* < 0.0001). The 46.0% of oocytes that are mature after eCG in midlife is not significantly different than the 40.2% of oocytes that are mature after eCG in young (Y + : M + , *P* = 0.2772). Therefore, eCG impedes maturation of ovulated oocytes and the effect of eCG on oocyte maturation is not affected by maternal age. Others have also reported ovulation of immature MI oocytes in rats and mice that have undergone superovulation with eCG [32–35]. Exogenous FSH also impedes maturation of up to 50% of oocytes retrieved from women after COS FSH stimulation [36–44].

This study used a highly purified eCG preparation with little contamination by LH or other components (AF Parlow, personal communication). However, the eCG molecule possesses some inherent LH activity, although in rodents the LH activity of eCG is significantly lower than that of hCG [45]. Given that both the control groups and the eCG treatment groups received hCG, the reduced % yields of viable and mature oocytes in the eCG treatment groups are most likely attributable to the FSH activity of eCG rather than its LH activity. Replication of these studies using an FSH without contaminating LH activity would provide confirmation.

Conventionally in IVF laboratories, ovarian oocytes retrieved with a PB1 are scored as mature MII oocytes, whereas those without a PB1 are scored as immature. However, the dynamic events that occur in the aftermath of oocyte maturation continue to unfold post-ovulation. In the hours after eCG administration and ovulation induced by hCG, first polar bodies in mouse oocytes undergo a process of degeneration and ultimately disappear. Thus, an “Alternative Explanation” for the lack of a PB1 is that oocytes lacking a polar body are actually mature MII eggs whose PB1 entirely degenerated and disappeared by the time of oocyte collection, due to PB1 degeneration induced by eCG. If this Alternative Explanation is correct, it would imply that oocytes lacking a PB1 are artifacts of eCG-induced PB1 degeneration, thereby discrediting the notion that eCG impedes oocyte maturation. Given the key importance of this question, we sought to determine which interpretation is most likely the correct one.

If eCG treatment impedes maturation, one would expect to retrieve a significant contingent of GV, GVBD, and/or prometaphase I oocytes (“Pre-MI oocytes”), as well as anaphase I, and/or telophase I oocytes (“Pre-MII oocytes”), along with oocytes with the morphology of MI and MII oocytes. However, if the Alternative Explanation is operative, then maturation of the oocyte population has already been completed, so the earlier meiotic phases have already been surpassed. In that case, the recovered oocytes will be almost entirely of the MII morphology, harboring condensed, aligned chromosomes and well-formed spindles, with or without a PB1; and without a significant contingent of GV, GVBD, anaphase or telophase oocytes.

**Fig. 3:**
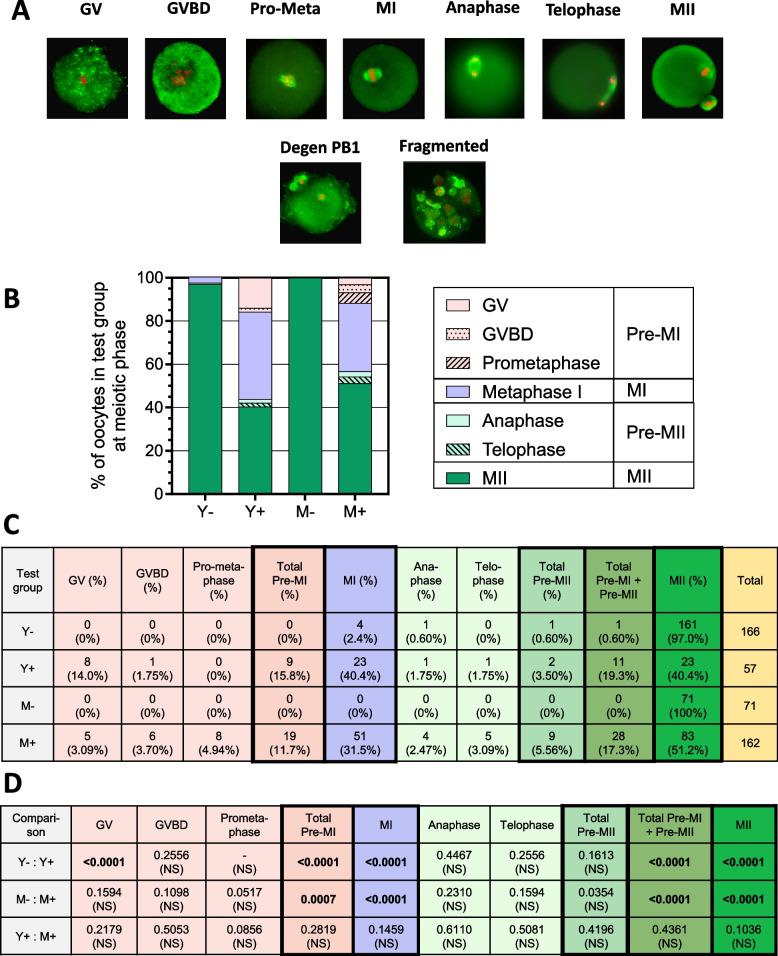
Morphologic evidence that eCG impedes oocyte maturation equally in young and midlife mice. **A **Fluorescence microscopy images of maturation and degenerative phases. Oocytes with stained chromosomes and spindles in the plane of view by fluorescence microscopy were scored for stage of meiotic progression as germinal vessel stage (GV); germinal vesicle breakdown (GVBD), pro-metaphase I (Pro-meta), meiosis I (MI), anaphase I, telophase I, or meiosis II (MII) (top row). Oocytes with a degenerating polar body (second row, left) and apoptotic fragmentation (right). **B **% of Y-, Y + *, M- and M* + *oocytes in each maturation phase (graph to left), with color coding indicated (to right).* **C **Numerical yields and percentages (%) of oocytes in each maturation phase. Color codes match those of the graph in 3B. **D** *P values comparing ratios of maturation phases to total oocytes for Y-: Y* + *, M-: M* + *and Y* + *: M* + *comparisons*. Bold P values are significant after one-sided Fisher exact and Benjamini–Hochberg tests, and un-bolded P values are not significant (NS). The P value for Y-:Y + was designated as “- (NS)” because both comparators had numerators with zero values

As shown in Fig. 3, just 0.6% and (0% of oocytes, collected from eCG-untreated young and midlife mice respectively, have Pre-MI or Pre-MII oocytes (-PB1). In contrast, 19.3% of oocytes collected from eCG-treated young mice and 17.3% from midlife were Pre-MI or Pre-MII oocytes. The increases in Pre-MI and Pre-MII oocytes in both the young and midlife test groups they are treated with eCG are extremely significant (Y-: Y + , *P* < 0.0001; M-: M + , *P* < 0.0001). For eCG-treated young and midlife groups combined, 17.8% of oocytes were in GV, GVBD, prometaphase, anaphase or telophase, compared to just 0.42% oocytes from untreated SAMP8, a 42.4-fold enrichment (*P* < 0.0001).

However, the Alternative Explanation presupposes that few to no oocytes in the eCG-treated test group (e.g., 0/219) will be observed at the earlier maturation phases (GV, GVBD, etc.). The difference between these two ratios is extremely significant (*P* < 0.0001). Therefore, the data support the notion that eCG impedes oocyte maturation.

Miao et al. quantified the timeline of polar body extrusion, degeneration, and ultimate disappearance from ovulated mouse oocytes after eCG stimulation and hCG ovulation induction [46]. They found that PB1 extrusion occurred between 8 and 10 h post hCG. They also found that the disappearance of the PB1 is a slow process in which a degenerating PB1 is an obligate-intermediate that acquires a rough, granular, or apoptotic morphology prior to its complete degeneration [46]. A low percentage of oocytes lose the PB1 between 12 and 16 h, with the PB1 still present, but in a degenerated state in 100% of MII oocytes by 20 h, and not completing its disappearance until 32 h post hCG. This is 12 h or more after commencement of post-ovulatory aging (POA), a COC morphology that is readily discernable in light microscopy by its abnormally expanded cumulus [16].

If the SAMP8 oocytes were actively maturing at the time of recovery 14–16 h post hCG, we’d expect a low % of oocytes with a PB1 to have a degenerated PB1 morphology; and we’d expect that few or no oocytes would have a POA morphology. If the Alternative Explanation is correct, then most or all MII oocytes would have a PB1 with a degenerating morphology, and most to all oocytes recovered, with or without a PB1, would have a POA morphology. Scoring of PB1 morphology in fluorescence microscopy found that only 2.56% (see Fig. 3A, Row 2 left frame for example) of MII SAMP8 oocytes from eCG-treated mice had a PB1 that was degenerating, far lower than upwards of 100% expected by the Alternative Explanation. Moreover, 317/317 (100%) of viable oocytes from eCG-treated SAMP8 mice and examined by light microscopy, both from young and midlife mice, had a freshly ovulated morphology, with 0/317 (0%) with a POA morphology. Taken together, the prevalence of pre-MI and pre-MII oocytes, the low % of MII oocytes harboring polar bodies with a degenerated morphology, and the lack of POA oocytes recovered from eCG-treated mice, are consistent with the conclusion that eCG impedes oocyte maturation.

The % of recovered oocytes from eCG-treated young and midlife mice in GVBD, Prometaphase I, MI, anaphase, telophase, and MII did not differ between young and midlife mice. However, young eCG-treated mice did have a significantly higher % of oocytes in the GV phase than midlife eCG-treated mice (Y + : 14.04% vs. M + : 3.09%, *P* = 0.0058). This may suggest that oocytes from young eCG-treated mice have a somewhat slower transition from GV to the more advanced stages of maturation. This difference does not appear to impact the overall advancement of maturation of oocytes from the Pre MI to MI or from Pre MII to MII, as there were no significant differences in distributions of these phases between the two groups (data not shown).

The notion that elevated FSH activity impedes oocyte maturation at the juncture of GV and GVBD is supported by prior published data of Upton et al., who reported that transgenic *in vivo* overexpression of FSH in mice causes a partial block at the GV-GVBD juncture that impedes resumption of meiosis [47]. Future studies employing a time course of eCG treatment in SAMP8 mice would determine whether eCG causes a blockade or a delay in maturation, and would define the duration of any such delay, and the maturation stage(s) at which the delay is occurring.

The results whereby 97 – 100% of ovulated oocytes recovered from naturally cycling SAMP8 mice are mature implies that strict regulatory mechanisms exist to permit ovulation only once oocytes have reached maturity. Given that immature oocytes do not give rise to offspring, this tight regulation would seem to be strongly selected for to avoid wastage of female gametes. However, the high percentage of oocytes that are ovulated at a variety of immature stages in eCG-treated SAMP8 mice suggests that eCG dysregulates these controls.

We postulate that eCG both impedes oocyte maturation *and* dysregulates controls that prohibit ovulation of immature oocytes. An alternative possibility, whereby eCG permits ovulatory *release* of immature oocytes without also impeding oocyte maturation, cannot be ruled out. Future time course studies that determine the maturation stages of peri-ovulatory ovarian oocytes vs. ovulated ampullary oocytes from eCG-treated and naturally cycling mice would test these notions.

### Different pathways are used for eCG gonadotropin ootoxicity and impeding of oocyte maturation

In related studies, the course eCG treatment in young and midlife mice was extended to include at 17 day time point (comprised of successive weekly eCG injections prior to hCG ovulation induction), in addition to the 0 and 2.6 day time points [15]. If the same molecular signaling pathways are used to modulate oocyte viability and maturation, one might expect a significant correlation between them throughout the time course. However, 2-tailed Spearman analyses shows a poor correlation (r = 0.4857; *P* = 0.3556; Supplementary Table S1). The poor correlation suggests that different pathways are used by eCG to modulate ootoxicity and impediment of oocyte maturation.

### Elevated FSH is pro-growth, pro-survival, and pro-maturation to oocytes in the pre-ovulatory period while being ootoxic and inhibitory to maturation in the ovulatory period

**Fig. 4:**
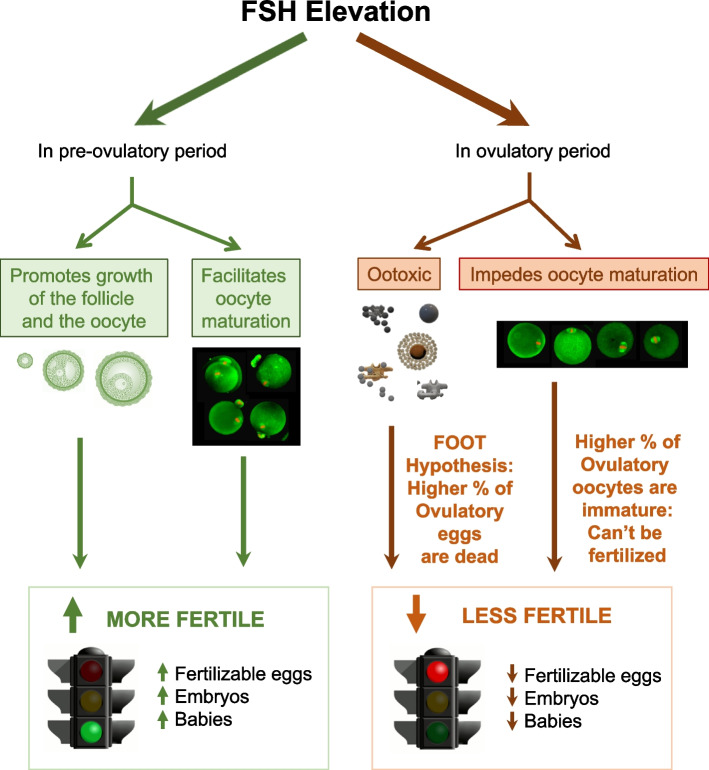
The FOOT Hypothesis and maturation inhibition by high FSH: *Paradoxical fertility effects of high FSH in preovulatory vs. ovulatory oocytes.* It is well known that high dose FSH in the preovulatory period promotes growth and facilitates maturation of the oocyte, both of which are essential to fertility (green, left side of Figure). Paradoxically, elevated FSH in the ovulatory period is ootoxic and impedes oocyte maturation, both of which are detrimental to fertility (orange and brown, right side of figure). The FSH OoToxicity Hypothesis—"FOOT Hypothesis”—states that high endogenous or exogenous FSH decreases fertility because it is toxic to ovulatory oocytes (brown arrows, middle of diagram). We speculate that high FSH in naturally cycling DOR women and COS/IUI patients also reduces fecundability by impeding maturation of a percentage of oocytes. Images of individual fluorescently stained oocytes were juxtaposed to images comprised of mature (left) and immature (right) oocytes, respectively. Attribution citations for Figures: Growing follicles (re-colored green), [48]; Red traffic light [49]; Green traffic light [50]

High FSH activity in the preovulatory period is a well-known pro-survival signal that promotes growth and maturation progression of pre-ovulatory oocytes in the ovary. However, we observe that high FSH activity in ovulatory oocytes is potently ootoxic and impedes oocyte maturation (Fig. 4). This poses an important paradox. Whereas the pathways by which FSH promotes oocyte survival, growth, and maturation of ovarian oocytes have received extensive study, mechanisms by which high FSH activity is ootoxic to ovulatory oocytes and impedes maturation are not yet understood and are worthy of investigation in future studies going forward.

### The FOOT Hypothesis (FSH OoToxicity Hypothesis)

We hypothesize that endogenous and exogenous FSH elevation are ootoxic to a significant % of ovulatory oocytes in AMA women, significantly reducing fertility in natural cycles, and reducing pregnancy rates in COS-IUI cycles, especially in AMA women. We call this the FSH-OoToxicity Hypothesis—the FOOT Hypothesis (Fig. 4). Given the mouse data in this paper and recent human studies showing that women undergoing COS cycles have lower rates of oocyte maturation than those undergoing natural cycle stimulation [51], we speculate that FSH elevation also impedes maturation and reduces pregnancy rates and fertility in COS/IUI and infertile naturally cycling DOR patients. Future studies would determine the extent to which high FSH may exert ootoxic and maturation-inhibitory effects on ovulated oocytes, reducing fertility in women with high endogenous FSH, and diminishing pregnancy rates in women attempting pregnancy via COS with exogenous FSH.

High FSH activity has other detrimental effects on fecundity. These include polyspermic fertilization [52], impedance of oviductal sperm transport and embryo transport [53, 54], reduced endometrial receptivity [55, 56], increased oocyte chromosome and spindle misalignments, and oocyte aneuploidy [3, 15, 47]. Moreover, estradiol elevation is induced by COS stimulation with high dose FSH, and prior studies have shown that estradiol elevation adversely impacts implantation, and imposes an ampullary tubal block that prevents transit of ova into the isthmus so they can enter the uterus [55, 57].

These many adverse effects of FSH along with ootoxicity and maturation inhibition might be multiplicative in their collective adverse impacts on fertility. They may help explain the very low pregnancy success rates of naturally cycling DOR (diminished ovarian reserve) and POI (primary ovarian insufficiency) women with high endogenous FSH, as well as COS patients attempting pregnancy by intrauterine insemination (IUI).

Might there be a selective advantage to the multiplicity of detrimental effects of high FSH? It is well known that high endogenous FSH occurs predominantly in women of advanced maternal age. In the pretechnological wild before the era of modern medicine, new AMA mothers had low chances of surviving to raise their children to adulthood; nor were they as likely to contribute to raising their grandchildren [58–60]. High endogenous FSH also increases the likelihood of multiple pregnancies, and mothers of multiples and their babies were also less likely to survive. It is logical to theorize that the multiple pathways by which high FSH decreases the likelihood of pregnancy paradoxically promote natural selection by *preventing* pregnancy in AMA DOR women.

### ActRIIB:Fc restores oocyte maturation blocked by gonadotropin elevation but not does not prevent gonadotropin ootoxicity to ovulated oocytes

**Fig. 5:**
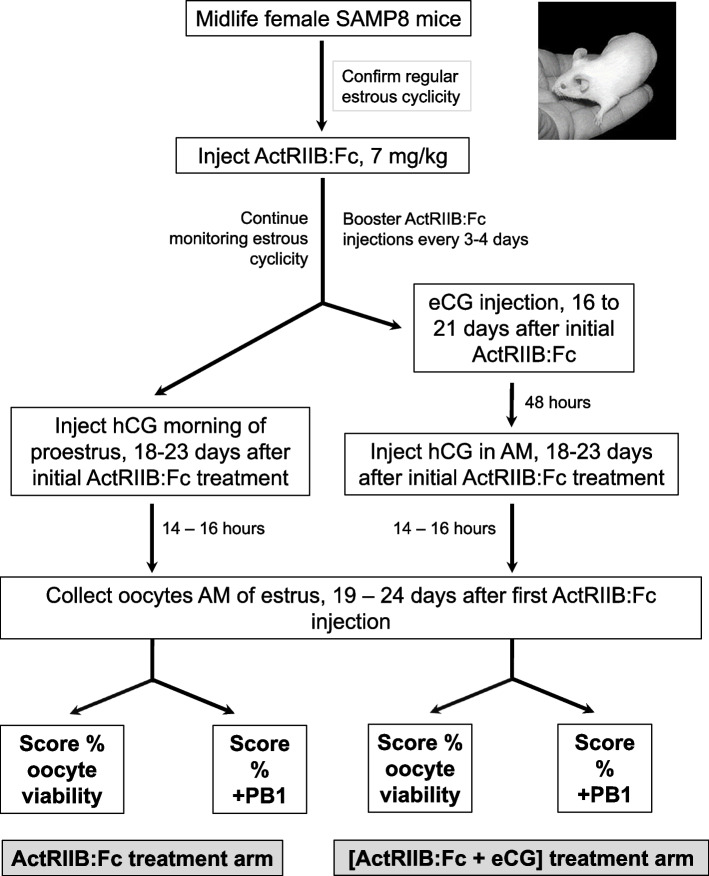
Treatment protocols for midlife SAMP8 mice treated with ActRIIB:Fc and with ActRIIB:Fc + eCG

Midlife SAMP8 mice have lower fertility than young SAMP8 mice [2]. Previously we reported that ActRIIB:Fc therapeutically increases the number of viable ovulated oocytes/mouse, reduces the percentage of ovulated oocytes with chromosome and spindle misalignments, and increases fertility of midlife SAMP8 mice [3]. Using the protocol in Fig. 5, we sought to determine whether co-treatment of eCG-treated midlife mice with ActRIIB:Fc therapeutically improves the percentage of ovulated oocytes that are recovered viable and/or the percentage that are mature.

**Fig. 6:**
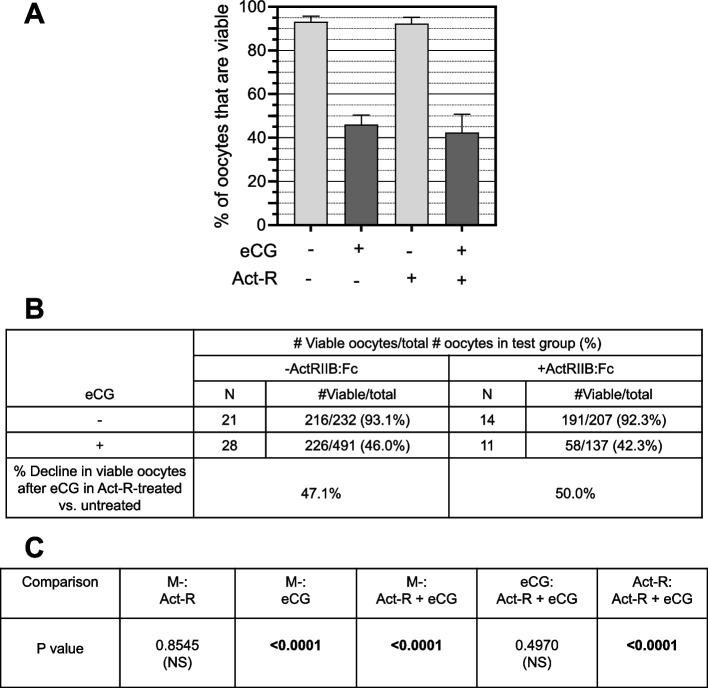
ActRIIB:Fc does not prevent eCG ootoxicity. **A** *Graphical comparisons of % viability rates*. X-axis, top row: “-”, no eCG; “ + ”, eCG-treated. X-axis, bottom row: “Act-R”: ActRIIB:Fc. “- “, Act-R untreated; “ + ”, Act-R-treated. Light gray bars: -eCG; dark gray bars: + eCG. **B** *% decline in viable oocytes*. % decline due to eCG treatment was computed for Act-R-untreated mice (left column) and Act-R-treated mice (right column) due to eCG treatment = 100*([# viable/# total oocytes for untreated mice]—[#viable/total oocytes for eCG-treated mice]). **C**. *P values for comparisons of #viable/# total oocytes between test groups*. M-, untreated; Act-R + eCG, treated with Act-R and eCG

Significant declines in viability are observed in eCG-treated test groups, irrespective of ActRIIB:Fc co-treatment (Fig. 6: Untreated mice 93.1%, vs. eCG-treated mice 46.0%: *P* < 0.0001; ActRIIB:Fc-treated 92.3% vs. ActRIIB:Fc + eCG treated 42.3%: *P* < 0.0001). The incremental percent decline in viable oocytes caused by eCG is 47.1% in ActRIIB:Fc-untreated mice and 50% in ActRIIB:Fc-treated mice, with no significant impact of ActRIIB:Fc on the % decline viable in oocytes caused by eCG (eCG treated, 46.0%, vs. eCG + ActRIIB:Fc, 42.3%: *P* = 0.4790 (NS)). While ActRIIB:Fc had no effect on the % of viable oocytes, its biological potency was confirmed by its lowering of endogenous estrus FSH levels in midlife mice after both 1—4 and 21 – 24 days of ActRIIB:Fc administration (*P* < 0.0001; Supplementary Table S2).

ActRIIB:Fc does not affect the % of viable oocytes in eCG-untreated mice (Untreated, 93.1% vs. ActRIIB:Fc-treated 92.3%: *P* = 0.8545 (NS); Fig. 6A-C). This is not inconsistent with our prior data showing that ActRIIB:Fc increases the number of viable oocytes/mouse, because ActRIIB:Fc commensurately increases the number of non-viable oocytes/mouse ([3]; Bernstein et al., manuscript in preparation). ActRIIB:Fc treatment of midlife mice has no impact on the maturation rate of ovulated oocytes from eCG-untreated midlife mice, with 97.0% of oocytes exhibiting maturity (+ PB1) without ActRIIB:Fc vs. 97.2% of oocytes exhibiting maturity after ActRIIB:Fc treatment (*P* = 1.0000).

**Fig. 7:**
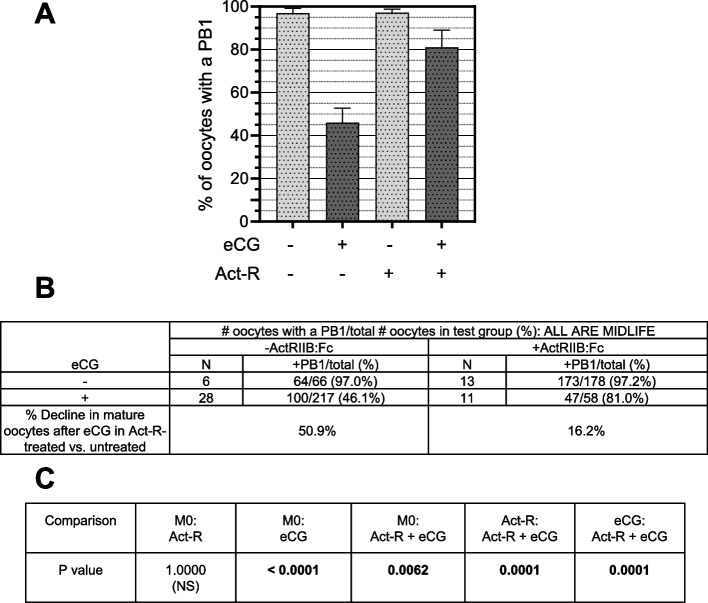
ActRIIB:Fc restores maturation of mouse oocytes that is blocked by treatment with eCG. **A** *Graphical comparisons of maturation rates*. X-axis, top row: “- “, no eCG; “ + ”, eCG-treated. X-axis, bottom row: “Act-R”: ActRIIB:Fc. Act-R untreated; “ + ”, Act-R-treated. Light gray stippled bars: -eCG; dark gray stippled bars: + eCG. **B** *% decline in mature oocytes*. % decline due to eCG treatment was computed for Act-R-untreated mice (left column) and Act-R-treated mice (right column) due to eCG treatment = 100*([# mature/# total oocytes for untreated mice]-[#mature/total oocytes for eCG-treated mice]). The *P* value is for the significance of the difference in % decline of mature oocytes after eCG between Act-R-untreated and Act-R-treated test groups. **C** *P values for comparisons of # mature/# total oocytes between test groups*

Whereas the maturation rate is reduced from 97.0% to 46.1% by eCG, co-treatment of eCG-treated mice with ActRIIB:Fc increases the rate of maturation to 81.0% (*P* = 0.0001; Fig. 7). Power analyses confirmed the sufficiency of samples sizes for this experiment. With 217 and 58 oocytes respectively in the eCG and Act-R + eCG groups being compared, the *P* value of 0.0001 is significant to 95% power with α = 0.01 in a two-sided Chi square test comparing two proportions. Whereas eCG in the absence of ActRIIB:Fc causes a 50.9% incremental decline in oocyte maturation, eCG-treated mice co-treated with ActRIIB:Fc display only a 16.2% decline in maturation rate. Overall, ActRIIB:Fc treatment restores 68.6% of oocyte maturation that is blocked by eCG.

### Pathways and therapeutics

Activin upregulates pituitary secretion of FSH. FSH is lowered in midlife mice by ActRIIB:Fc during the several weeks long period of treatment (Supplementary Table S2). 5 IU eCG has FSH activity of sufficient potency that it would be expected to overcome.

ActRIIB:Fc’s lowering of endogenous FSH. Therefore, we expected that mice in the Act-R + eCG group experience lowered FSH activity during the first 18 -21 days of ActRIIB:Fc treatment, followed by raised FSH activity the last 2.6 days before oocyte harvest. The inability of ActRIIB:Fc to prevent eCG ootoxicity is consistent with the notion that high FSH activity *per-se* during the final 2.6 days before retrieval is a cause of eCG ootoxicity.

It is noteworthy that ActRIIB:Fc significantly improves oocyte maturation rates even though FSH activity is expected to be quite elevated in the last 2.6 days before oocyte recovery. Several alternative explanations are possible: (1) ActRIIB:Fc’s lowering of FSH during the several weeks *before* eCG administration is responsible for restoring oocyte maturation; (2) ActRIIB:Fc interferes with signaling steps downstream of activin induction of FSH production that would otherwise impede oocyte maturation; (3) ActRIIB:Fc promotes oocyte maturation by suppressing activin functions that are independent of FSH and it’s signaling pathway. Further studies of the mechanisms by which FSH, activin, activin receptor, and their functional interactions may modulate oocyte maturation will help elucidate these pathways.

10—50% of oocytes that are recovered in human IVF regimens are immature at the time of retrieval [36] [38–44] [37]. Given that immature oocytes cannot be fertilized, diminished yield of mature oocytes stands as an impediment to IVF success, especially for the contingent of patients with low oocyte maturation rates [61]. Of the stimulated IVF/ICSI cycles performed in 2022, Benadiva and colleagues found that 41% (335/825) of all cycles had fewer than 75% mature oocytes, with these patients experiencing an average maturation rate of only 58% (Drs. Claudio Benadiva and Alison Bartolucci, U. Conn Health Center, personal communication). This likely imposes a substantial incremental reduction in pregnancy success rates in a significant percentage of patients that is important to therapeutically address.

*In vitro* maturation (IVM) has shown some promise in addressing this issue, although recent improvements in yields of mature oocytes have been rather modest. Some improvements to first polar body extrusion rates have been achieved by supplementation of IVM media with antioxidants melatonin, N-acetyl cysteine (NAC), mogroside V, putrecscine, β-cryptoxanthin, peroxiredoxin-4, and resveratrol [62–68]. Oocyte maturation rates *in vitro* were also improved by myostatin (alias GDF8) and bone morphogenic protein-15 (BMP-15), both of which are members of the TGFβ superfamily [69, 70]. Increased rates of PB extrusion were also observed in IVM media supplemented with brain-derived neurotrophic factor, all-*trans* retinoic acid, leptin, kisspeptin, AY 9944-A-7 (which promotes intrafollicular accumulation of follicular fluid meiosis activating sterol), and growth hormone [71–76].

Fewer treatments have had proven beneficial effects on oocyte maturation *in vivo*. The proportion of mature oocytes collected in a human patient population with a history of high % of immature oocytes retrieved in IVF was improved using a dual ovulation trigger of GnRHa + hCG, compared to hCG alone [61]. Other treatments that improve rates of mammalian oocyte maturation *in vivo* include growth hormone, melatonin [77, 78], and now ActRIIB:Fc. Given the improvement from 46 to 81% rates of maturation after ActRIIB:Fc treatment of eCG-stimulated SAMP8 mice, activin modulators may hold therapeutic potential to improve both human and animal assisted reproduction by increasing the percentage of oocytes/cycle that are mature in COS IUI and IVF patients undergoing FSH stimulation.

In studies that link growth hormone and activin signaling, activin blocked induction of growth hormone mRNA stability and expression in cultured grass carp pituitary cells, whereas follistatin promoted growth hormone mRNA expression [79]. Given that follistatin and ActRIIB:Fc both bind activins to block their biological potency, and given that both growth hormone and ActRIIB:Fc both improve oocyte maturation rates *in vivo*, we speculate that ActRIIB:Fc, like follistatin, operates upstream of growth hormone, inducing pituitary growth hormone mRNA expression, which then increases oocyte maturation. Future studies will be required to test this hypothesis.

### Paradox of activin induction of oocyte maturation *in vitro* vs. ActRIIB:Fc induction of oocyte maturation *in vivo*

Prior studies by Itoh et al. and by Alak et al. demonstrated that activin A *stimulates* oocyte maturation [80–82]. This is paradoxical given that ActRIIB:Fc, a potent *antagonist* of activin activity, *induces* oocyte maturation. How to resolve this paradox by which agents that increase and agents that inhibit activin activity both appear to promote oocyte maturation?

Whereas the Itoh and Alak studies were performed *in vitro* by treating cultured COCs with activin, the current study was performed *in vivo* by treatment of whole animals with ActRIIB:Fc. The milieu of the oocyte is very different in the vitro and *in vivo* settings. The COCs used to perform the studies *in vitro* are comprised of an oocyte surrounded by a layer of granulosa cells. In this *in vitro* context, 2-way granulosa-oocyte interactions orchestrate oocyte growth and maturation. In contrast, the growing follicle as it exists *in vivo* also has a layer of thecal cells that surrounds the granulosa layer. In the *in vivo* context, growth and maturation of the follicle and the oocyte are orchestrated via tripartite theca-granulosa-oocyte interactions.

Thecal cells biosynthesize testosterone (T). Testosterone from thecal cells potently promotes oocyte maturation *in vitro* and *in vivo* [83, 84]. Activin A blocks StAR and HSD3B-driven thecal catalysis of T biosynthesis [85]. Thus, activin might be expected to block oocyte maturation *in vivo* where there are thecal cells, but it would not be expected to block maturation *in vitro*, since thecal cells are not present.

Activin has a second pathway by which it regulates oocyte maturation. This pathway *induces* oocyte maturation. Activin A promotes *in vitro* oocyte maturation by inducing cumulus granulosa cell mRNA expression and biological activity of lysyl oxidase (LOX) enzyme, an enzyme that catalyzes crosslinking of elastin and collagen fibers within the follicular extracellular matrix (ECM; [7] and references therein, [86–88]). However, whereas activin increases Lox mRNA and enzymatic activity, the increase in LOX activity was comparatively modest [86]. We speculate that *in vivo*, the net biological effect of ActRIIB:Fc-sequestration of activin is to prevent activin’s blockade of thecal testosterone biosynthesis, raising testosterone and thereby augmenting oocyte maturation. Future studies are needed to test the validity of these notions.

Ligands of the TGFβ superfamily and their receptors are promiscuous in their binding to one another, creating complexity in pinpointing the downstream signaling cascades that they regulate [89–91] and references therein. Activin A binds diverse receptors, including the ALK 4/7 receptor (Type 1), ActRIIA, ActRIIB, and BMPRII. ActRIIB:Fc binds to Activin A, GDF-8 and GDF-11, and with lower affinity to BMP-2 and BMP-7 [91]. Thus, an alternative explanation for why Activin A and ActRIIB:Fc treatments give paradoxical results may be that activin A and ActRIIB:Fc modulate different TGFβ signaling pathways with opposing downstream biological effects on oocyte maturation.

Goebel et al. have recently engineered ligand trap reagents that can potentially be used to target diverse TGFβ signaling pathways with great specificity (e.g., activins vs. GDFs vs. BMPs, etc.; [92]). Future studies employing novel ligand trap reagents and/or antibodies that target and inactivate specific activin, GDF and BMP pathways will be performed to pinpoint the aberrant signaling pathways and ultimately target them to therapeutically improve rates of oocyte maturation in patients undergoing assisted reproduction. Given that activin ligand trap ActRIIB:Fc restores oocyte maturation impeded by FSH but does not prevent ootoxicity, we propose that development of a “Hormone Normalization Therapy” (“HNT”; [3]  [93]) to restore FSH levels in DOR women to healthy young levels throughout the menstrual cycle may hold promise both for abrogating maturation inhibition and preventing FSH ootoxicity to ovulatory oocytes to increase fertility and pregnancy rates in DOR women.

## Conclusions

High FSH activity of eCG is ootoxic to ovulatory oocytes and it inhibits oocyte maturation. Midlife mice are more susceptible than young mice to FSH ootoxicity, while being equally susceptible to maturation inhibition. Administration of ActRIIB:Fc, which binds endogenous activin and inactivates its biological activity, restores most oocyte maturation blocked by the elevated FSH activity of eCG. Restoration of oocyte maturation by ActRIIB:Fc contradicts the previously accepted notion that activin promotes oocyte maturation.

We propose the FOOT Hypothesis for FSH ootoxicity: that high FSH from endogenous and exogenous sources is ootoxic to ovulatory oocytes, and is a previously unrecognized driver of infertility and poor reproductive outcomes in naturally cycling DOR patients and COS IUI patients, especially AMA women. Given the oocyte maturation data in animals and humans showing inhibition of oocyte maturation by high FSH activity, we speculate that endogenous FSH elevation also contributes to reduced fertility and pregnancy rates in naturally cycling DOR patients, and in COS/IUI patients. Development of HNT and ligand trap molecules that pinpoint specific TGFβ pathways may hold promise for therapeutic improvement of oocyte viability and maturation rates in these infertile patients.

### Supplementary Information

Supplementary Material 1.

## Data Availability

No datasets were generated or analysed during the current study.

## Abbreviations

Act-R: ActRIIB:Fc-treated
AMA: Advanced maternal age
BDM: Brunner, Dette and Munk
B-H: Benjamini-Hochberg
BMP: Bone-morphogenic protein
BMP-15: Bone-morphogenic protein-15
COC: Cumulus-oocyte complex
COS: Controlled ovarian stimulation
DOR: Diminished ovarian reserve
eCG: Equine chorionic gonadotropin
FOOT Hypothesis: FSH OoToxicity Hypothesis
FSH: Follicle stimulating hormone
GDF: Growth and differentiation factor
GV: Germinal vesicle phase
GVBD: Germinal vesical breakdown phase
hCG: Human chorionic gonadotropin
HNT: Hormone normalization therapy
HPO axis: Hypothalamic-pituitary-ovarian axis
IUI: Intrauterine insemination
IVM: *In vitro* Maturation
LOX: Lysyl oxidase
Metaphase I: MI
Metaphase II: MII
percentage: %
POA: Post-ovulatory aging
PB1: Polar body 1
POI: Primary ovarian insufficiency
Pro-meta: Prometaphase
SAMP8: Senescence-accelerated mouse prone-8
TGFβ: Transforming growth factor beta
