# Supplementary material for: Gonadotropin elevation is ootoxic to ovulatory oocytes and inhibits oocyte maturation, and activin decoy receptor ActRIIB:Fc therapeutically restores maturation
Source: Reprod Biol Endocrinol. 2024 May 6;22:52. doi: 10.1186/s12958-024-01224-8 (PMC11071334; doi:10.1186/s12958-024-01224-8)
Supplement: Supplementary file 1 — Supplementary Material 1. [file 12958_2024_1224_MOESM1_ESM.docx]

***Supplementary Materials***

**Legends for Supplementary Tables**

**Supplementary Table S1: Lack of correlation between % oocyte viability and % of oocytes with a PB1 suggests that different pathways are used by eCG to cause ootoxicity vs. impede oocyte maturation**. Y, young; M, midlife. Y0 and M0: eCG-untreated; Y2.6 and M2.6: eCG treated for 2.6 days before harvest; Y17 and M17: eCG treated for 17 days before harvest.

**Supplementary Table S2: ActRIIB:Fc is biologically active, significantly lowering serum FSH levels in regularly cycling midlife mice**. N, number of mice per study group. Sera were generated from bleeds collected the morning of estrus 0, 1-4 and 19 – 24 days after initiation of ActRIIB:Fc treatment.

**Supplementary Table S1**

| Test group of mice | % oocytes that are viable (#viable/total) | % of oocytes with a PB1  (# withPB1/total) |
| --- | --- | --- |
| Y0 | 93.4% (225/241) | 100.0% (77/77) |
| Y2.6 | 73.8% (144/195) | 40.2% (41/102) |
| Y17 | 56.3% (152/270) | 73.4% (94/128) |
| M0 | 93.1% (216/232) | 97.0% (64/66) |
| M2.6 | 46.0% (226/491) | 46.1% (100/217) |
| M17 | 31.9% (106/332) | 74.5% (79/106) |
| Significance of Correlations, Spearman Analyses  r = 0.4847; 2-sided P value, P = 0.3556 | | |

**Supplementary Table S2**

| Days ActRIIB:Fc | 0 | 1 - 4 | 21 - 24 |
| --- | --- | --- | --- |
| N, # mice | 8 | 9 | 9 |
| Mean FSH, pg/ml | 39,151 | 6,938 | 22,099 |
| SEM | 1,956 | 1,647 | 3,429 |
| % Inhibition | -- | 82.3% | 43.6% |
| P value vs. untreated | -- | <0.0001 | 0.0002 |
| Overall P value | <0.0001 | | |
